# Core–shell particles and monolithic columns; tools for simultaneous LC analysis of avanafil, sildenafil, apomorphine, trazodone, yohimbine, tramadol and dapoxetine in pharmaceutical dosage forms, counterfeit products and human plasma†

**DOI:** 10.1039/c9ra08717f

**Published:** 2020-01-08

**Authors:** Adel Ehab Ibrahim, Hisham Hashem, Magda Elhenawee, Hanaa Saleh

**Affiliations:** Pharmaceutical Analytical Chemistry Department, Faculty of Pharmacy, Port-Said University Egypt pharmacist_adel_2005@yahoo.com +20 1112332345; Pharmaceutical Analytical Chemistry Department, Faculty of Pharmacy, Zagazig University Egypt

## Abstract

By 2025, it's estimated that 322 million males worldwide will suffer from sexual disorders. This can give an estimation for the size of the pharmaceutical and counterfeit products industry for the next few years. Meanwhile, green analytical chemistry forced itself to decrease the massive environmental pollution and hence new analytical methodologies are needed to replace the old ones that consume large amounts of hazardous solvents. In this research, two new methods were validated for determination of seven recognized drugs used in treatment of male impotence, premature ejaculation as well as enhancing sexual libido by HPLC on RP-C18 core–shell particulate and monolithic columns. The study was extended to compare the capabilities of those stationary phases to accommodate greener chromatography concepts without loss of efficiency. Both morphologies shortened the analysis time relative to the previously reported conventional HPLC methods by different approaches. Core–shell particles had higher efficiency in terms of theoretical plates' number and enhanced resolution power which enabled lower detection limits. However, the monolithic column had lower column backpressure which enabled the use of ethanol as a greener alternative solvent at even higher flow rates. The methods were finally applied successfully for the determination of drugs under study in pharmaceutical dosage forms, counterfeit products and in human plasma.

## Abstract 2

By 2025, it's estimated that 322 million males worldwide will suffer from sexual disorders. This study provides two simple green tools for analysis of some male sexual enhancers using HPLC on core–shell particles and monolithic RP-columns.

## Graphical abstract

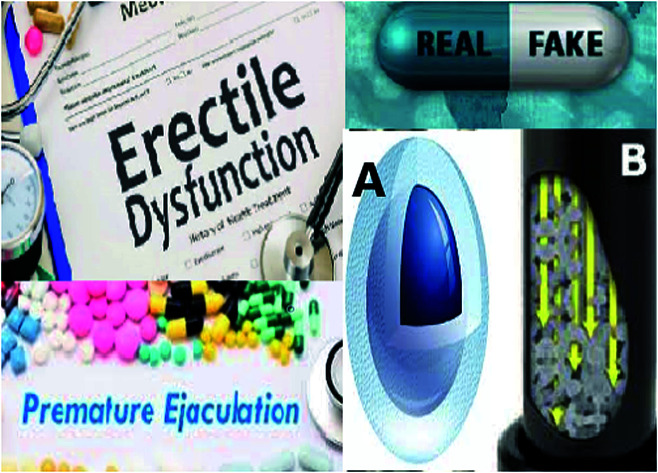

## 1. Introduction

The prevalence of male sexual disorders is noticeably huge and has been increasing during the past few years. Sexual disorder prevalence is expected to rise above 320 million males worldwide by the year 2025.^1^ Erectile dysfunction (ED) and premature ejaculation (PE) are the most frequent and main sexual dysfunctions. ED is defined as the inability to maintain erection during sexual intercourse, while PE is defined as ejaculation prior to male desire. About 20–40% of males over 60 years old suffer ED and this figure continues to rise as age increases.^2^ Sexual disorders are not confined to elder patients, but recently pervade youth. The main causes for male sexual disorders are classified into organic and psychogenic factors. There are organic factors such as hypertension, diabetes mellitus, smoking, *etc.*, however psychological factors are becoming of high risk due to the negative effect of different stresses in people's life recently. ED & PE have been known to co-occur in 30% of patients.^3^ Co-administration of drugs used in their treatments is predicted and future combination regimens may exist. Meanwhile, counterfeit medication for treatment of such sexual disorders has been developing globally, especially in the developing countries.^4^ Herbal products received special interest by patients for their low impact on human health. However, adulteration of such informal products due to lack of regulatory supervision resulted in falsified products containing active ingredients.

The seven drugs under study are used to treat male sexual disorders. Avanafil (AVN), sildenafil (SDN), apomorphine (APM) and yohimbine (YHB) are recognized drugs in treatment of male impotence. Tramadol (TMD) and trazodone (TZD) are drugs abused in treatment of premature ejaculation. Dapoxetine (DPX) is the first drug to be approved for on-demand treatment of premature ejaculation. TZD and YHB were reported also to enhance sexual libido. SDN and AVN are phosphodiesterase-5 (PDE-5) inhibitors used in treatment of ED. SDN was the first drug approved as PDE-5 inhibitor by the United States Food and Drug Act (US FDA), while AVN is a new ultra short acting PDE-5 inhibitor.^5^ APM is a dopaminergic agonist (D1 and D2) that can be used as sublingual tablets to treat ED.^6^ YHB is a natural alkaloid that was reproduced synthetically because of its α2-adrenoceptor antagonist effect which was found to enhance ED with psychological origin.^6^ TMD is a strong opioid analgesic that has much lower dependence than morphine. It has side effect of delaying ejaculation so it was abused in treatment of PE,^7^ beside its effect in mode elevation. DPX is the first short acting drug approved mainly for on-demand treatment of PE. DPX is a selective serotonin reuptake inhibitor (SSRI). TZD is a unique drug that is abused in treatment of ED and PE, simultaneously. TZD is a serotonin reuptake inhibitor so it delays ejaculation. It also causes a condition known as priapism which is a condition in which a penis remains erect for hours in the absence of stimulation or after stimulation has ended so it was also tried for treatment of ED.^8^ Another added value for TZD was reported to increase sexual libido in both men and women.^9,10^

The stationary phase is the main core where separation of different analytes takes place. The choice of stationary phase became one of the main and most critical steps in the development of LC methods because it requires compromise between separation efficiency, analysis time and environmental safety. Lots of analyses are done daily by research and industrial quality control laboratories which must serve high through output, so the time required for analysis is a critical parameter. Environmental safety and greening the analytical methodologies is another important consideration. Conventional LC equipment can generate around half liter of organic solvents daily,^11^ that's why LC is targeted by green chemistry concepts.

Older trends for enhancing separation efficiency depended on decreasing stationary phase particles size; however this was limited by the high column backpressure developed. To shorten analysis time, shorter columns were used, but this affected separation efficiency. Another means was to increase flow rates, but it was counteracted also by the developed column backpressure. Greener chromatography focuses on decreasing the consumption of organic solvents and/or replacing environmentally toxic solvents by safer ones.

In the years of 2003 and 2004, sub-2 μm particles and ultra performance liquid chromatography (UPLC) were introduced successfully on commercial level. UPLC reduced analysis time, lowered solvent consumption and enhanced sensitivity and separation efficiency. However, it's not a widespread technique in economical laboratories due to the short chromatographic column lifetime, low reproducibility and high maintenance costs required which arise from the high backpressure developed on the UPLC system.^12^ So, the new stationary phase geometries, core–shell particles and silica monolith, became a promising tool for the purpose of economic, simple, green and efficient methodologies in short analysis time. The ideas of these stationary phase morphologies are old. Several papers described and compared the conventional totally porous reversed phase particles to these new morphologies.^13–15^ However, new trends in developing efficient, economical and faster, together with greener analysis methods utilized these new stationary phase morphologies on the conventional HPLC systems.

Literature survey revealed that individual determination of the drugs under study was reported extensively. Some papers were reported for simultaneous determination of drugs used in treatment of ED^16–18^ and some others were reported for simultaneous determination of some antidepressant drugs that can be abused in treatment of PE.^19,20^ However, as discussed before, the co-administration of drugs for treatment of ED and PE is possible. Few methods were reported for co-determination of two drugs used for ED and PE such as SDN/DPX,^21,22^ AVN/DPX,^23,24^ SDN/TMD^25^ and SDN/YHB.^26^ To the best of our knowledge, only one method was reported for simultaneous determination of three PDE-5 inhibitors and four SSRIs, and was applied on pharmaceutical dosage forms.^27^

This study provides a new comparison tool between monolithic rods and core–shell 2.7 μm particles columns and their capabilities to be used for greener chromatography. The study covers the areas of column's efficiency, selectivity, analysis time, and solvent consumption. Also, the proposed study offers two new highly efficient methods that were successfully validated for the identification, separation and quantification of seven drugs used in treatment of male sexual disorders for the first time. The methods were applied successfully for identification and determination of these drugs in pharmaceutical dosage forms, counterfeit products and human plasma.

## 2. Experimental

### 2.1. Materials

Sodium octane sulfonate, orthophosphoric acid, sodium hydroxide and ethylenediamine tetracetic acid disodium salt (EDTA) were analytical grade and were purchased from Merck, Germany. Ethanol (EtOH), acetonitrile (ACN) and methanol (MeOH) were HPLC grade and were purchased from J.T. Baker, Netherlands. De-ionized water was freshly prepared in-house by Millipore water purification system.

**Fig. 1:**
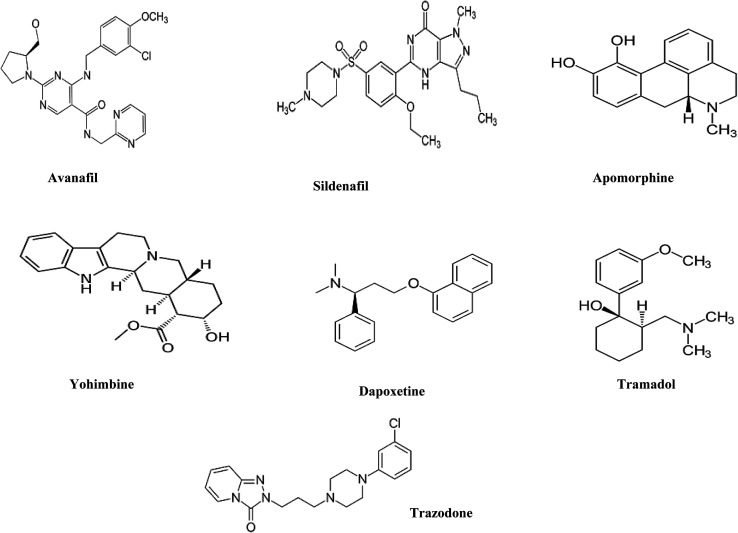
Chemical structures of the seven male sexual enhancer drugs under study.

AVN and DPX as hydrochloride salts were kindly supplied by Al-Andalus pharmaceutical company, Egypt. SDN as citrate, APM, TMD, TZD and YHB, all as hydrochloride salts, were kindly supplied by EIPICo., Egypt. All drugs were pharmaceutical grade (chemical structures Fig. 1).

Pharmaceutical dosage forms; Vigor forte® chewable tablets (batch number: 61212), and Trittico® tablets (batch number: 1401046) were kindly supplied by EIPICo, Egypt. Yohimbex® tablets (batch number: 11051), Joybox® tablets (batch number: 171436) and Tramundin® tablets (batch number: 153385) were purchased from Egyptian market. Erovanafil® tablets (batch number: 160367) was kindly supplied by Al-Andalus pharmaceutical company, Egypt (for composition refer to Section 3.1.4). Counterfeit products were purchased from the illegal Egyptian market under names; Tiger-King®, Fox®, Big-P®, and Love-zone®.

Blank human plasma samples were purchased from the Egyptian national blood bank.

### 2.2. Equipment

All experiments were done on Agilent HPLC series-1200 composed of a solvent pump model-G1311A, auto-sampler model-G1329A, connected to column compartment model-G1316A and UV detector model-G1314A from Agilent technologies, Germany.

pH meter (model-713) from Metrohm industries, Switzerland was used to adjust pH during the study.

Two chromatographic columns were used in the proposed study. Monolithic Chromolith® Performance RP-18e (100 × 4.6 mm) column was purchased from Merck, Germany. Core–shell, Poroshell® EC-C18 2.7 μm (150 × 4.6 mm) column was purchased from Agilent technologies, Germany.

### 2.3. Chromatographic conditions

Mobile phase-A (buffer) consists of 0.02 g EDTA and 2.16 g sodium octane sulfonate dissolved in 1000 mL water then adjust pH to 3.5 using 3 M orthophosphoric acid solution. Mobile phase B is HPLC grade EtOH or ACN (Table 1).

Gradient elution programming for the proposed methods under study

**Table:** 

Column I	Monolithic Chromolith®	
Flow rate	2 mL min^−1^	
Mobile phase gradient programming	Mobile phase A (buffer)	Mobile phase B (ethanol)
0.0–6.0 min	65.0%	35.0%
6.0–8.0 min	65.0–40.0%	35.0–60.0%
8.0–10.0 min	40.0%	60.0%

a For determination of drugs in plasma, run time 12–18 min.

**Table:** 

Column II	Core–shell Poroshell®	
Flow rate	1 mL min^−1^	
Mobile phase gradient programming	Mobile phase A (buffer)	Mobile phase B (aetonitrile)
0.0–11.0 min	65.0%	35.0%
11.0–12.0 min	65.0–35.0%	35.0–65.0%
12.0–16.0 min^a^	35.0%	65.0%

All analyses were done by gradient elution technique at the flow rates as listed in Table 1. Auto-sampler injection volume was 20.0 μL and column compartment was kept at 35 °C. Detection was done at 210 nm using UV-detector.

### 2.4. Stock solutions, calibration standards and quality control samples

Stock solution of AVN, APM, TMD, SDN, TZD, YHB and DPX was prepared in solvent mixture (MeOH : H_2_O, 1 : 1) at concentrations 5 mg mL^−1^.

The working solutions were prepared by dilution of the stock solution using the solvent mixture to prepare calibration standards at concentrations 2.0, 10.0, 25.0, 50.0, 75.0 and 100.0 μg mL^−1^. The quality control (QC) samples were prepared at three different concentrations, low (QCL), medium (QCM) and high (QCH) which were 5.0, 50.0 and 80.0 μg mL^−1^ by spiking a freshly prepared placebo solution. Placebo solution was prepared by dispersing 10.0 g of commonly used excipients per liter water (*e.g.* starch, magnesium stearate, titanium dioxide, sodium starch glycolate and carboxymethyl cellulose sodium). All stock solutions, calibration standards and QC samples were stored in amber containers in a refrigerator at 2–8 °C.

For determination of drugs in plasma, linearity calibration standards were prepared at concentrations 1.0, 10.0, 20.0, 30.0, 40.0 and 50.0 μg mL^−1^ by diluting the stock solution in blank plasma samples then vortex mixed for 10 minutes. According to FDA guidelines, the total organic solvent added should not exceed 2% of biological sample.^28^ QC samples were prepared in four concentrations at 2.5, 5.0, 25.0 and 40.0 μg mL^−1^ by dilution in blank plasma samples and vortex mixed in the same way. All calibration standards and QC samples were stored in freezer (−20 °C).

### 2.5. Sample preparation

Ten tablets from each studied dosage form and counterfeit product, were weighed, ground and mixed. Stock solutions were prepared by dissolving the average weight corresponding to one tablet of each product in 100.0 mL solvent mixture. Stock solutions were placed in a sonicator for 10.0 min then filtered.

For Trittico®, Tramundin®, Joybox® and Erovanafil® tablets, the working solutions were prepared by dilution of 5 mL of the filtrate to 100.0 mL using the solvent mixture. For Vigor forte® and Yohimbex® tablets, the working solutions were prepared by dilution of 20.0 mL of the filtrate to 100.0 mL using the solvent mixture. Working solutions of counterfeit products' tablets were prepared directly by dissolving the average weight of one tablet in 200.0 mL solvent mixture, sonicated for 10.0 min then filtered.

Frozen plasma samples were thawed before analysis at room temperature. An aliquot of 400 μL of calibration standards and QC samples were added in 1.5 mL Eppendorf centrifuge tube then 600 μL of ACN was added. The mixture was shaken on vortex mixer for 5 min then centrifuged at 10000 rpm for another 10.0 min. 20 μL of the clear supernatant was injected on the HPLC-UV system.

### 2.6. Method validation

Method validation was performed for the quantitative determination of the seven drugs to evaluate the proposed methods on both stationary phases. Validation was done according to FDA^28^ and ICH guidelines.^29^

## 3. Results and discussion

### 3.1. Method validation

#### 3.1.1. Selectivity

**Fig. 2:**
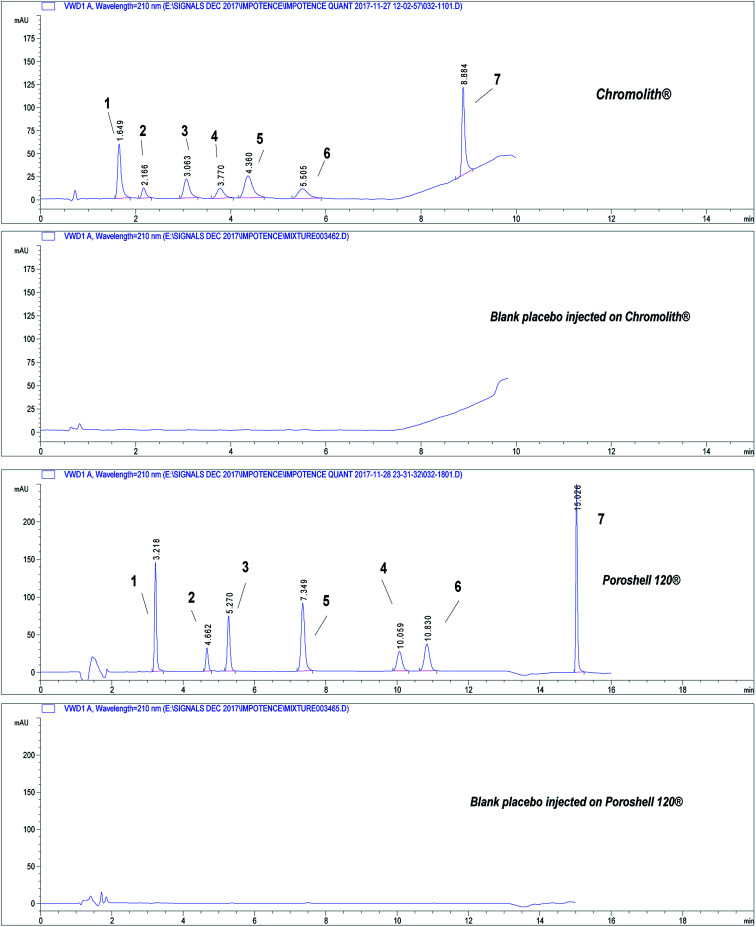
Chromatograms showing separation of the seven drugs under study on core–shell and monolithic columns. Chromatographic conditions: refer to Table 1, sample QCM at concentration 50.0 μg mL^−1^. (1) APM, (2) TMD, (3) YHB, (4) AVN, (5) TZD, (6) SDN, (7) DPX.

Separation of the seven drugs was achieved on both stationary phases at good resolution between their peaks. Chromatograms in Fig. 2 show separation done on both columns under study for QCM samples spiked in freshly prepared placebo solution as shown in injected blank placebo chromatograms. Excipients in the tablet dosage forms and counterfeit products did not interfere.

**Fig. 3:**
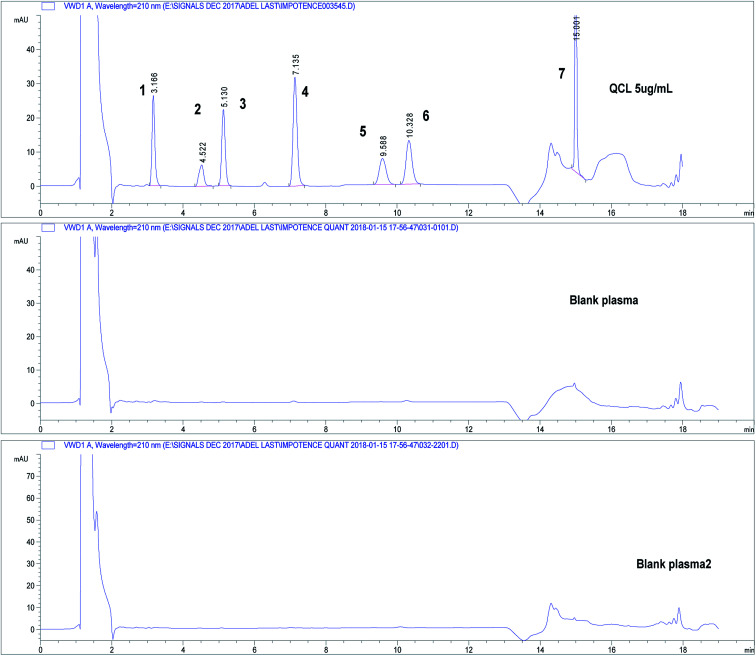
Chromatograms showing separation of drugs under study in spiked human plasma. Chromatographic conditions: refer to Table 1, sample QCL at concentration 5.0 μg mL^−1^. Column: core–shell Poroshell® EC-C18, 2.7 μm. (1) APM, (2) TMD, (3) YHB, (4) TZD, (5) AVN, (6) SDN, (7) DPX.

Three independent blank human plasma samples from different human sources were treated and injected on the HPLC-UV system and were compared to spiked plasma samples to test interferences from any endogenous substances. Fig. 3 shows separation of drugs using QCL sample (5.0 μg mL^−1^) spiked in blank human plasma. Independent blank human plasma samples were injected and no interference from any endogenous material that may be found in plasma was detected.

#### 3.1.2. Linearity

Six concentrations of each drug within the specified range were prepared as described in placebo and human plasma and injected in triplicates. The calibration curves were constructed by plotting the area of peaks corresponding to each drug against its concentration. Regression data showed very good linearity within the specified range (ESI Tables 1 and 2†).

Limits of detection (LOD) and quantification (LOQ) were calculated according to signal-to-noise ratios; (10 : 1) for LOQ and (3 : 1) for LOD. LODs and LOQs as shown in (Table 2) indicated the sensitivity of the proposed methods.

Chromatographic performance results obtained on core–shell and monolithic columns for determination of drugs under study

**Table:** 

Parameter	Chromolith® Performance RP-18e						
	APM	TMD	YHB	AVN	TZD	SDN	DPX
Average *t*_R_ (minutes)^d^	1.7 ± 0.2	2.2 ± 0.2	3.1 ± 0.0	3.8 ± 0.4	4.4 ± 0.2	5.6 ± 0.0	8.9 ± 0.2
Selectivity, *K*	—	1.6	1.6	1.2	1.2	1.31	1.71
Resolution	—	3.8	4.9	2.9	2.1	3.3	13.9
Symmetry factor	0.6 ± 0.4	0.7 ± 0.8	0.7 ± 0.6	0.8 ± 0.4	0.7 ± 0.6	0.7 ± 0.8	0.6 ± 0.8
*N* a ^,^ d	2800.0 ± 1.2	3300.0 ± 2.0	3300.0 ± 0.6	3200.0 ± 1.8	3200.0 ± 0.6	3400.0 ± 1.2	—e
*P* b	120 bar						
LOD ng mL^−1^	100.0	500.0	200.0	200.0	100.0	200.0	100.0
LOQ ng mL^−1^	400.0	1000.0	600.0	600.0	400.0	500.0	400.0
Total run time	9.5 minutes						
Volume of organic solvent consumed^c^	8.8 mL per run + 1.2 mL per re-equilibration						

a Number of theoretical plates per 100 mm long stationary phase, for QC-sample at concentration 5 μg mL^−1^ of each drug.

b Column backpressure at zero time.

c Volume consumed per chromatographic run.

d Results = average ± relative standard deviation.

e The comparison is irrelevant in the gradient elution region of the chromatogram.

**Table:** 

Parameter	Poroshell 120® EC-C18 2.7 μm						
	APM	TMD	YHB	TZD	AVN	SDN	DPX
Average *t*_R_ (minutes)^d^	3.2 ± 0.0	4.7 ± 0.2	5.3 ± 0.0	7.2 ± 0.4	9.8 ± 0.2	10.5 ± 0.2	15.0 ± 0.2
Selectivity, *K*	—	1.8	1.2	1.6	1.4	1.2	1.4
Resolution	—	12.9	4.6	12.7	12.0	2.9	23.5
Symmetry factor	0.8 ± 0.2	0.8 ± 0.6	0.8 ± 0.4	0.8 ± 0.6	0.8 ± 0.2	0.8 ± 0.4	0.8 ± 0.8
*N* a ^,^ d	11 200.0 ± 1.2	15 200 ± 1.6	15 100.0 ± 0.8	14 700.0 ± 0.8	15 500.0 ± 2.2	16 900.0 ± 1.8	—e
*P* b	200 bar						
LOD ng mL^−1^	50.0	200.0	100.0	50.0	100.0	50.0	50.0
LOQ ng mL^−1^	200.0	500.0	300.0	200.0	300.0	200.0	200.0
Total run time	16.0 minutes						
Volume of organic solvent consumed^c^	7.0 mL per run + 1.8 mL per re-equilibration						

#### 3.1.3. Accuracy and precision

Accuracies of the proposed methods were established across the specified ranges by analyzing QC samples prepared at different concentration levels. QC samples were injected in triplicates and the average recovery percentages were calculated. Repeatability and intermediate precisions were determined in the same day (intra-day) and on three different days (inter-day) using QC-samples prepared by spiking placebo solution or plasma as clarified in paragraph 2.4. Accuracy and precision results indicated the trueness and reproducibility of the analytical procedures (ESI Tables 1 and 2†).

#### 3.1.4. Robustness

Both methods were tested for minor variations in the chromatographic conditions. Column temperature was changed at increments of 2 °C at (33.0, 35.0 and 37.0 °C), percentage of organic solvent in mobile phase at zero time (34.0, 35.0 and 36.0%) and buffer pH (3.4, 3.5 and 3.6). These minor variations did not cause significant effect on separation efficiency as a function of theoretical plates (*N*) or recovery percentage as seen from results in (ESI Tables 3 and 4†).

#### 3.1.5. Analytical application

The methods were applied for determination of the seven drugs in their different marketed pharmaceutical dosage forms; Vigor-forte® chewable tablets, Trittico®, Yohimbex®, Joybox®, Tramundin® and Erovanafil® all as tablets (composition and results are listed in Table 3). The results show that the specified amounts of active pharmaceutical ingredients are within the acceptable limits and results were comparable on both columns. The method was also applied to check adulteration of counterfeit products by any of the drugs under study using the validated methods. The studied counterfeit products; Tiger-King®, Fox®, Big-P®, and Love-zone® are labeled to be of herbal origin. The obtained results are shown in (Table 3); some of the drugs under study were found and quantified. The obtained results show that the four studied counterfeit products are falsified products marketed within the illegal Egyptian market under the claim of being of herbal origin. However, under the proposed experimental conditions, they didn't show peaks that may correspond to any other ingredients extracted. The identification of the presence of the drugs was easily confirmed from the retention time on the two different columns and using different polarities of the mobile phase by simply changing the organic modifier since the aqueous phase was the same.

**Table:** Analysis of dosage forms and counterfeit products on columns under study

Dosage-form/counterfeit product	Labeled amount	Chromolith® found amount^a^	Poroshell® found amount^a^
Vigor-forte®	APM 3 mg	100.8 ± 3.8	100.8 ± 5.4
	SDN 50 mg	96.8 ± 2.2	94.0 ± 1.6
Trittico®	TZD 100 mg	99.2 ± 3.2	96.4 ± 3.0
Joybox®	DPX 60 mg	96.0 ± 1.6	100.8 ± 1.6
Tramundin®	TMD 100 mg	92.6 ± 1.4	99.0 ± 0.6
Yohimbex®	YHB 5 mg	95.2 ± 3.0	96.4 ± 3.0
Erovanafil®	AVN 100 mg	92.0 ± 1.2	91.4 ± 1.0
Tiger-King®^b^	TMD	10.2 mg per tablet ± 3.2	—
	SDN	20.8 mg per tablet ± 2.0	
	DPX	20.6 mg per tablet ± 1.4	
Fox®^b^	SDN	20.4 mg per tablet ± 1.4	—
	DPX	30.4 mg per tablet ± 2.8	
Big-P®^b^	SDN	25.2 mg per tablet ± 1.2	—
Love-zone®^b^	SDN	55.8 mg per tablet ± 1.6	—
	DPX	30.2 mg per tablet ± 2.8	

a Results = recovery% ± RSD.

b Counterfeit products have no labeled data for drugs under study; they are supposed to be of natural herbal origin.

### 3.2. Core–shell *versus* totally porous particles

Two different chromatographic methods were developed for determination of seven drugs that are being used in treatment of male sexual disorders using two promising stationary phase technologies. The same aqueous buffer was used on both columns. ACN was used on core–shell particles column because of the high backpressure developed on the column when EtOH was used (Table 2). Monolithic rods exhibited higher permeability so can accommodate the high viscosity of water/EtOH mobile phase and also can withstand higher mobile phase flow rates leading to shorter analysis time (Table 2). EtOH is known as a biodegradable and cheap solvent, and hence is considered greener than ACN, which is widely used organic solvent used in LC due to its unique characters (*e.g.* lower viscosity in mixture with water, very low chemical reactivity and low UV cut-off). On relating ACN to environmental safety, it was found to be more dangerous than EtOH. The incineration of ACN produces (NO_2_) gas. This gas is known to be responsible for acidic rains. Also synthesis of ACN requires higher energy than other solvents.

The use of EtOH instead of ACN slightly affected the order of elution of drugs under study for AVN which has switched its elution order with TZD (Table 2). This may be attributed to the different polarities of EtOH and ACN. Since elution of analytes in RPLC depends on difference of partitioning between the mobile and the stationary phases, it seems like AVN has higher partitioning into EtOH/buffer mobile phase than into ACN/buffer mobile phase.

On the other hand, core–shell particles exhibited higher separation efficiency in terms of resolution and selectivity. Number of theoretical plates was higher and peaks symmetry was better in case of core–shell particles. Moreover, the LOD and LOQ were lower on core–shell than on monolithic column which enhanced sensitivity of the method and this may be attributed to the better peak symmetry (Table 2). It should be noted that *N* was compared for peaks eluted within the time of isocratic elution only, because it's irrelevant to compare *N* for peaks within the gradient elution period (DPX peak only).

Analysis was shorter on monolithic column (less than 9.0 min), while core–shell particles required double that time (16.0 min) due to its shorter length and ability to withstand higher flow rates. To re-equilibrate the columns between chromatographic runs, a mobile phase volume equivalent to 5 times the void volume (*V*_M_) is required for washing. Despite having shorter length, monolithic column has larger *V*_M_ than core–shell column, so monolithic column requires larger re-equilibration volume. But since the flow rate on silica monolith is double that on core–shell particles, the final re-equilibration time between runs was found to be nearly the same on both columns due to difference of applied flow rates (4.0 & 5.0 min on monolithic and core–shell columns, respectively).

Both methods were found to be economical since the total volume of organic modifiers consumed per chromatographic run was minimal, taking in consideration the higher flow rate used on monolithic column and the better environmental safety of EtOH than ACN (Table 2). The shorter analysis time means lower solvent consumption which is safer for environment and also more economical.

### 3.3. Evaluation of the analytical procedure

**Fig. 4:**
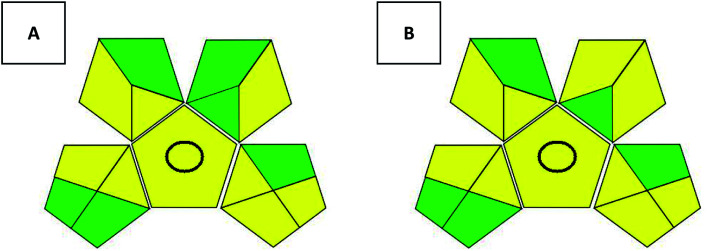
Evaluation of the proposed methods using GAPI pictograms (method A: ethanol used on monolithic column, method B: acetonitrile used on core–shell column). In GAPI, any red zone represents high ecological impact, yellow zones represent lower impact and green zones represent safe effect to environment.

Several tools have been introduced recently as green methods metrics. Since claiming greenness is not enough, such metrics consider several factors to evaluate newly introduced methodologies and compare them to the old methods. The National Environmental Methods Index (NEMI) was first introduced, and then it was followed by several metrics including the analytical eco-scale and the Green Analytical Procedure Index (GAPI).^11^ GAPI is a promising metric that considers all the steps involved within the analytical procedure including sample preparation, reagents and solvents used, instrumentation required and finally waste production and disposal. GAPI^30^ comprises 5 pentagrams containing 15 zones covering the whole analytical procedure. It uses a code of 3 colors in which the green represents low harm to the environment, then yellow and red symbolize medium and high ecological risks, respectively. The two proposed methods were evaluated on GAPI (Fig. 4). GAPI pictograms for both methods don't include red zones. Both methods utilize less than 10.0 mL organic solvent, however, EtOH use has the superiority of less health hazard than ACN so it show one more green zone than method linked to ACN.

## 4. Conclusion

In our study we compared performances of monolithic RP-rods with core–shell 2.7 μm particles for simultaneous determination of seven drugs used in enhancing male sexual performance. Some of the studied drugs can be used simultaneously as adulterants in counterfeit products which had been proved. Combination of therapies for PE and ED is now produced as single dosage form. Both columns under study exhibited very good separation of the seven drugs. Monolithic column enabled the use of higher flow rates for faster analysis time and also enabled the use of ethanol as greener organic modifier. However, core–shell particles proved higher separation efficiency, lower detection and quantification limits. The methods can be applied using the conventional HPLC instruments found in quality control and research laboratories without the need for special instrumentation as that required for UPLC. Besides, the longer lifetime and lower price of our columns compared to sub-2 μm columns encourages economical pharmaceutical industry laboratories to use them as replacement for the sub-2 μm UPLC columns.

Two simple fast LC methods were validated for the simultaneous determination of apomorphine, sildenafil, avanafil, yohimbine, trazodone, tramadol and dapoxetine and applied successfully for their determination in the corresponding dosage forms, in plasma and in checking the adulteration of counterfeit products. The overall analysis time didn't exceed 9.0 and 16.0 min. on monolithic and core–shell columns, respectively.

## Conflicts of interest

The authors declare that there is no conflict of interest. This article doesn't contain any studies with animals or human volunteers.

## Supplementary Material

RA-010-C9RA08717F-s001
