# Supplementary material for: Core–shell particles and monolithic columns; tools for simultaneous LC analysis of avanafil, sildenafil, apomorphine, trazodone, yohimbine, tramadol and dapoxetine in pharmaceutical dosage forms, counterfeit products and human plasma
Source: RSC Adv. 2020 Jan 8;10(3):1379–87. doi: 10.1039/c9ra08717f (PMC9047519; doi:10.1039/c9ra08717f)
Supplement: RA-010-C9RA08717F-s001 [file RA-010-C9RA08717F-s001.pdf]

Electronic Supplementary Material (ESI) for RSC Advances.
This journal is © The Royal Society of Chemistry 2019

Supplementary material table 1: Linearity and precision results for determination of drugs on core-shell and monolithic columns

Drug	Linearity equation	Regression R^2	Intra-day repeatability (Recovery%± RSD*)			Inter-day intermediate precision (Recovery%± RSD*)			Column
			QCL	QCM	QCH	QCL	QCM	QCH	
APM	Y=66.4X+31.8	0.999	97.0± 1.0	100.2± 0.4	100.2± 0.2	95.0± 1.4	99.4± 0.4	100.0 ±0.2	Chromolith® Performance RP-18e
TMD	Y=15.4X+7.4	0.999	93.0±1.2	99.4±0.4	99.8±1.0	94.2±1.0	99.4±0.4	99.4±0.4	
YHB	Y=39.0X+22.6	0.999	96.6±2.8	99.8±0.2	100.0±0.5	95.6±1.0	99.2±0.6	100.2±0.4	
AVN	Y=25.6X-0.0	0.999	93.6±1.4	98.5±0.2	98.4±0.6	95.4±5.0	98.2±0.6	98.2±0.2	
TZD	Y=65.0X+59.8	0.999	93.8±1.8	101.2±0.2	102.2±0.8	94.2±1.2	101.2±0.2	102.0±0.8	
SDN	Y=35.8X+5.6	0.999	98.6±2.4	99.4±0.4	100.2±1.4	97.2±1.8	99.6±0.4	98.2±0.4	
DPX	Y=80.8X+53.2	0.999	93.6±4.6	101.1±0.4	99.6±0.6	94.4±1.2	101.4±1.1	99.6±0.2	
APM	Y=117.0X+250.4	0.994	100.8±1.4	103.8±0.4	99.6±1.4	98.6±1.0	104.4±0.2	100.2±0.4	Poroshell 120® EC-C18 2.7µm
TMD	Y=28.4X+24.8	0.998	95.0±0.6	100.3±0.2	99.2±0.2	91.4±3.8	100.4±0.8	99.6±0.8	
YHB	Y=71.4X+115.2	0.997	99.4±1.8	101.2±0.8	99.0±0.2	96.4±2.4	99.8±0.6	98.2±0.8	
AVN	Y=51.2X+22.6	0.998	95.9±3.0	99.2±0.2	93.0±2.0	94.8±2.0	98.4±1.4	93.8±1.8	
TZD	Y=125.8X+142.2	0.996	93.0±0.8	99.8±0.4	98.2±1.4	96.2±2.0	100.4±0.8	98.4±1.6	
SDN	Y=70.6X+93.0	0.998	93.6±0.6	99.0±0.2	98.4±0.6	95.2±1.2	99.0±0.4	98.4±0.6	
DPX	Y=133.4X+515.0	0.994	98.4±1.0	106.8±0.2	100.2±0.4	97.2±1.2	106.8±0.4	99.8±0.6	

*** RSD (%Relative standard deviation)**

Supplementary material table 2: Linearity and precision data for determination of drugs under study in plasma

Drug	Linearity equation	Regression R^2	Intra-day repeatability (Recovery%± RSD*)				Inter-day intermediate precision (Recovery%± RSD*)			
			QCL1	QCL2	QCM	QCH	QCL1	QCL2	QCM	QCH
APM	Y=7.4X+30.3	0.976	90.8±5.0	101.2±1.2	108.0±2.0	91.0±1.4	92.4±5.0	102.4±3.0	106.6±2.4	91.0±1.6
TMD	Y=11.8X-2.4	0.999	103.6±1.2	107.6±0.6	104.6±0.2	101.0±0.4	101.6±2.2	106.0±4.0	105.2±4.0	100.6±1.0
YHB	Y=31.8X-5.6	0.999	95.2±0.8	106.4±0.8	103.6±0.2	101.6±0.4	96.4±1.6	104.2±3.4	103.2±0.2	100.0±2.4
TZD	Y=54.0X+11.8	0.999	91.6±1.6	105.8±1.2	104.6±0.4	102.0±0.2	91.1±4.8	104.6±1.4	104.6±0.6	100.6±2.6
AVN	Y=20.8X-5.4	0.999	102.4±0.4	99.2±1.4	105.2±1.0	100.4±0.2	104.4±2.2	97.0±4.0	105.0±0.8	100.8±3.6
SDN	Y=32.6X-8.0	0.999	90.2±4.2	105.8±1.0	103.6±0.4	102.2±0.2	90.8±2.8	103.8±2.2	102.6±0.6	100.0±2.6
DPX	Y=64.0X-13.6	0.999	94.0±0.5	105.8±0.6	103.0±0.6	102.6±0.2	92.4±6.0	106.4±2.6	102.2±0.4	100.0±3.0

*** RSD (%Relative standard deviation)**

Supplementary material table 3: Robustness results of the proposed method on core-shell and monolithic columns
***Results were obtained for injections of QC-sample at concentration 50.0µg/mL of each drug**

Chromolith® performance RP-18e						
**Analyte**	**Changes in**					
	**Buffer pH (3.4, 3.5 and 3.6)**		**%Organic solvent (34, 35 and 36%)**		**Temperature (33, 35 and 37°C)**	
	**N***	**% Recovery***	**N**	**% Recovery**	**N**	**% Recovery**
**APM**	2700.0 ±2.4	99.8± 1.2	2700.0 ±3.8	98.6 ± 3.2	2700.0 ±1.8	99.0 ±1.2
**TMD**	3300.0 ± 2.2	98.6±2.2	3300.0 ± 2.4	98.6 ±2.4	3200.0 ± 2.4	99.2 ±1.2
**YHB**	3300.0 ± 1.4	100.0 ±1.8	3200.0 ± 3.2	98.8 ±1.6	3300.0 ± 1.8	98.6 ±1.4
**AVN**	3200.0 ± 3.2	98.6 ±1.2	3200.0 ± 2.2	98.4 ±2.4	3100.0 ± 2.2	99.0 ±1.2
**TZD**	3200.0 ± 1.8	100.0±1.8	3100.0 ± 2.0	100.0 ±3.2	3300.0 ± 1.1	100.2 ±1.0
**SDN**	3300.0 ±2.0	99.0 ±1.6	3200.0 ±2.2	98.6 ±2.4	3300.0 ±1.2	100.2 ±1.4
**DPX**	111000.0±2.4	100.4 ±2.0	111700.0 ±2.8	100.6 ±2.0	108600.0 ±1.0	100.4 ±1.8
**Poroshell 120® EC-C18 2.7µm**						
**APM**	11000.0 ± 7.6	103.0±1.8	11000.0 ± 2.8	102.4 ±2.2	11200.0 ± 3.6	102.0 ±1.4
**TMD**	15500.0 ±1.6	100.6±2.0	15000.0 ± 2.4	99.8 ±1.4	14900.0 ± 2.2	99.6 ±1.0
**YHB**	15000.0 ±2.2	99.0±1.6	14900.0 ± 2.4	99.6 ±1.4	15400.0 ± 1.8	100.0 ±1.8
**AVN**	15100.0 ± 3.4	98.6±2.8	15300.0 ± 3.4	98.8 ±1.0	15100.0 ± 1.2	99.0 ±0.8
**TZD**	15000.0 ± 2.6	99.8±1.2	14500.0 ± 2.2	100.0 ±0.8	14600.0 ± 2.6	100.8 ±1.2
**SDN**	16000.0 ± 3.2	99.8±1.4	16400.0 ± 3.2	98.4 ±2.2	16000.0 ± 1.6	100.2 ±0.8
**DPX**	341200.0 ± 3.0	105.2±1.6	340500.0 ± 3.0	104.4 ±1.8	334400.0 ± 2.8	105.8 ±1.4

*** Results = Average ± %relative standard deviation**

Supplementary material table 4: Robustness results of the proposed method for determination of drugs under study in plasma

Analyte	Changes in					
	Temperature (33, 35 and 37°C)		%ACN (34, 35 and 36%)		Buffer pH (3.4, 3.5 and 3.6)	
	N*	% Recovery*	N	% Recovery	N	% Recovery
**APM**	7400.0 ±2.6	101.6 ±1.8	7500.0 ±3.6	100.8 ±4.6	7400.0 ±1.0	104.6 ±3.0
**TMD**	4300.0 ±4.0	108.0 ±3.0	4200.0 ±15.2	103.4 ±1.2	4100.0 ±6.4	106.6 ±2.0
**YHB**	13100.0 ±1.6	106.6 ±0.8	12700.0 ±3.2	102.2 ±3.0	12900.0 ±0.8	106.4 ±0.8
**AVN**	18600.0 ±2.4	100.0 ±0.6	18200.0 ±1.4	94.8 ±5.2	18400.0 ±1.0	99.8 ±1.4
**TZD**	11700.0 ±0.6	106.2 ±0.2	11000.0 ±5.8	107.4 ±3.2	11800.0 ±1.4	105.8 ±1.0
**SDN**	17100.0 ±1.4	105.6 ±1.2	16700.0 ±4.4	101.6 ±3.6	17400.0 ±2.4	105.4 ±0.8
**DPX**	487600.0 ±0.6	111.0 ±2.8	481200.0 ±3.4	105.0 ±4.8	499500.0 ±2.6	106.4 ±1.4

***Results were obtained for injections of QCL-sample at concentration 5µg/mL of each drug**
*** Results = Average ± %relative standard deviation**
